# Supplementary material for: Effect of localization on the stability of mutualistic ecological networks
Source: Nat Commun. 2015 Dec 17;6:10179. doi: 10.1038/ncomms10179 (PMC4703855; doi:10.1038/ncomms10179)
Supplement: Supplementary Information — Supplementary Figures 1-17, Supplementary Tables 1-3, Supplementary Methods and Supplementary References. [file ncomms10179-s1.pdf]

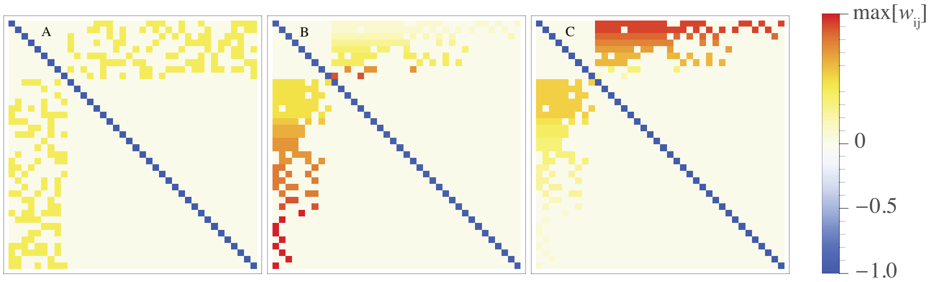

Supplementary Figure 1: **Parametrization of the interaction matrix**. The parametrization of the interaction matrix $w_{ij} = \gamma_0 B_{ij}/k_i^\delta$ allow us to consider different scenarios: A) Mean field case ($\delta = 0$) where there is no trade-off between species mutualistic strength and species degree; B) AInteraction strength-degree trade-off ($\delta = 0.5$), where a weak w_{ij} term is associated with a strong w_{ji} term (e.g. a strong dependency of the plant on the pollinator and a weak dependency of the pollinator on the plant); C) Positive strength-degree relation ($\delta = -0.5$), where we set a positive correlation between species interaction strength and degree.

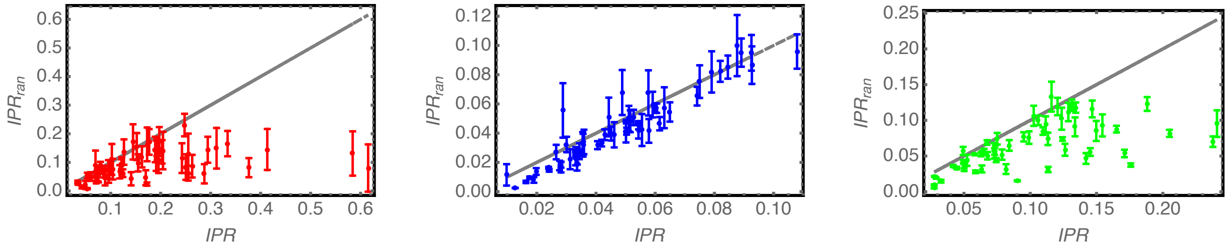

Supplementary Figure 2: **Localization in mutualistic networks: positive strength-degree relation.** Comparison between IPR ($\mathbf{v}_1$ in red, $\mathbf{u}_1$ in blue and $\mathbf{w}_H$ in green) of empirical pollinator networks with respect to null model 1 (1000 realization) for $\delta = -0.5$.

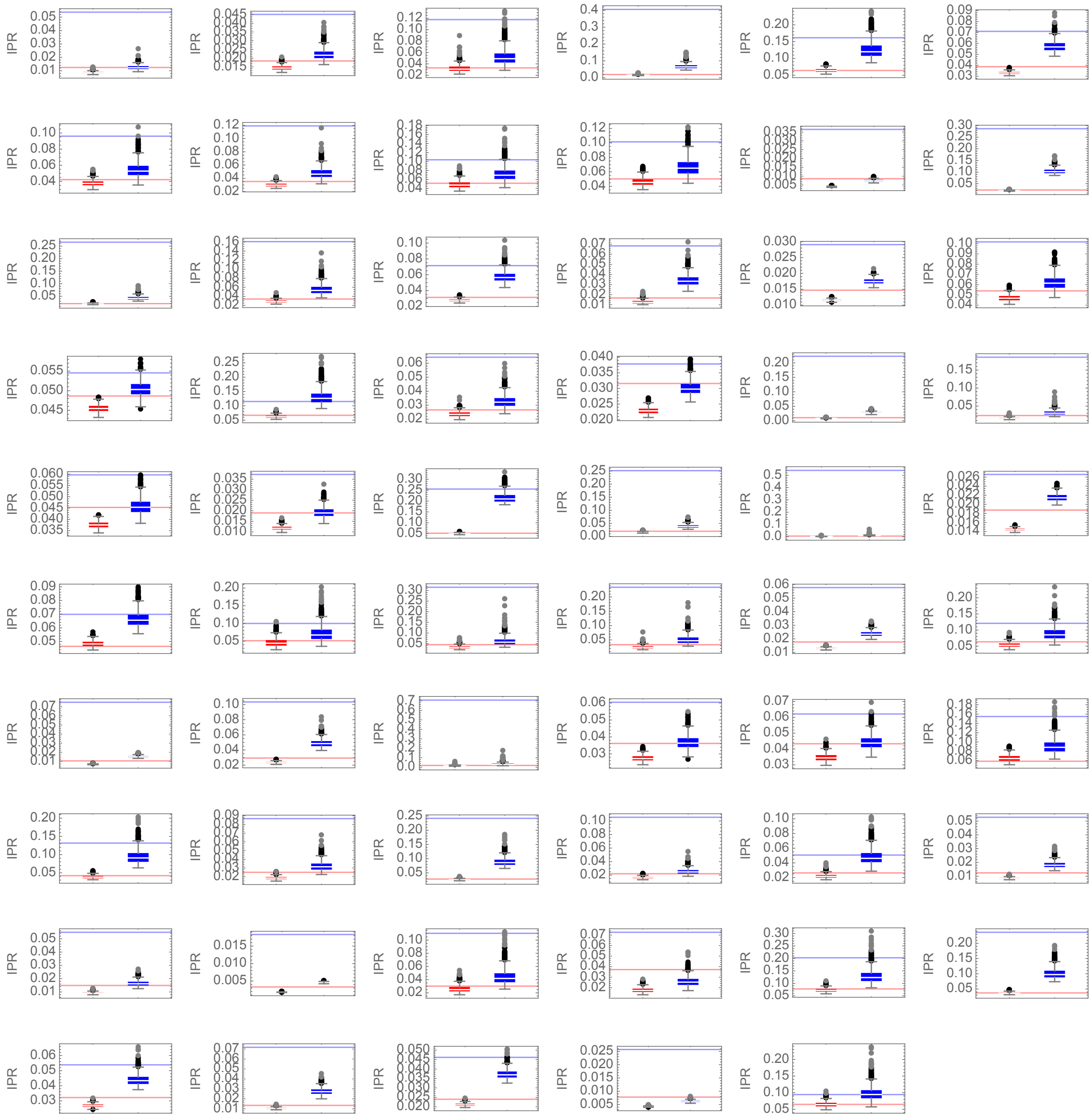

Supplementary Figure 3: **Localization in mutualistic networks: strength-degree trade-off.** Comparison between IPR ($\mathbf{v}_1$ in red, $\mathbf{u}_1$ in blue) for each of the 59 empirical pollinator networks (horizontal solid lines), and Box-Whisker plots for the IPR of the 1000 networks generated through the null model 1 ($\delta = 0.5$).

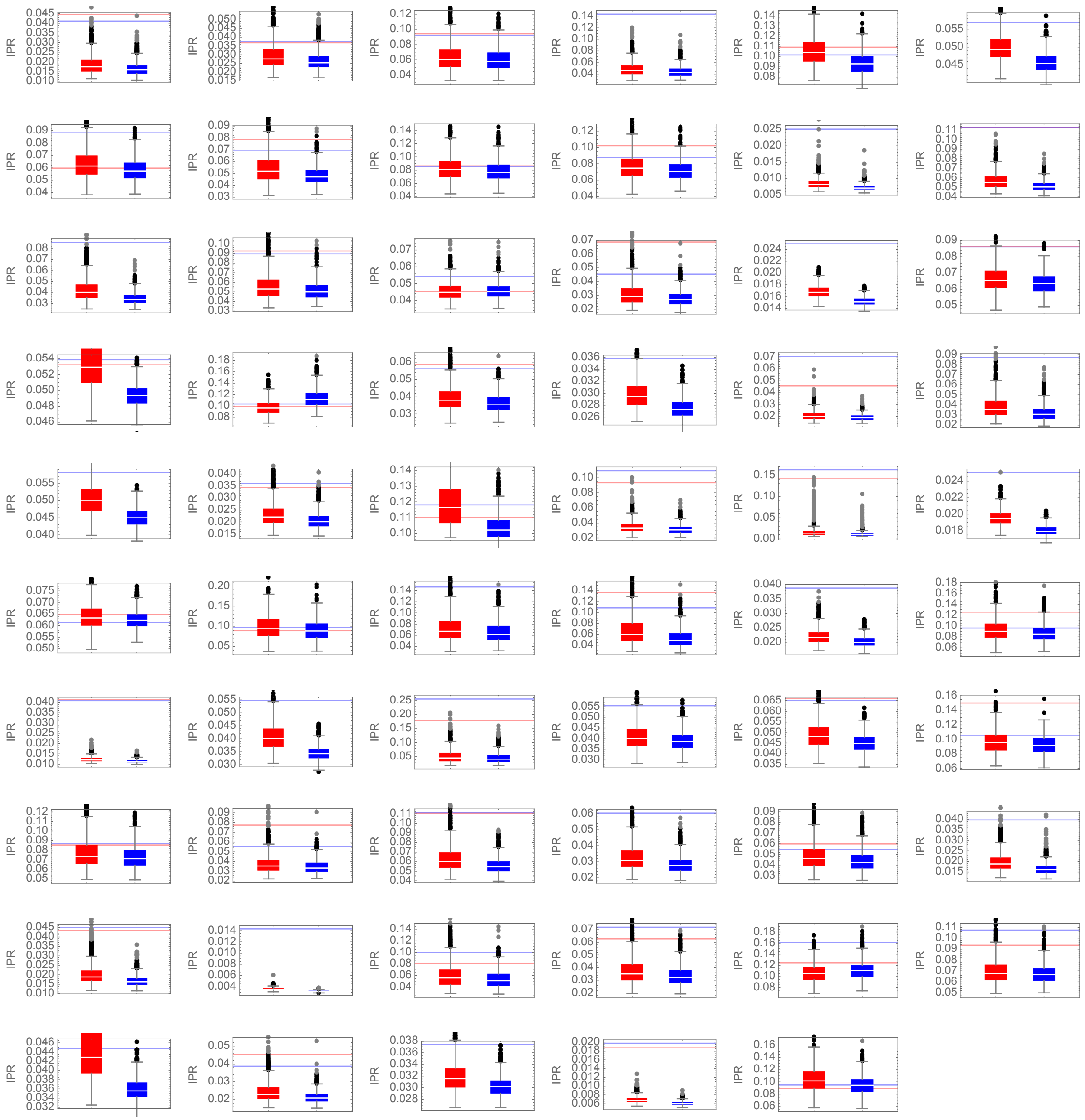

Supplementary Figure 4: **Localization in mutualistic networks: mean field case.** Comparison between IPR ($\mathbf{v}_1$ in red, $\mathbf{u}_1$ in blue) for each of the 59 empirical pollinator networks (horizontal solid lines), and Box-Whisker plots for the IPR of the 1000 networks generated through the null model 1 ($\delta = 0$).

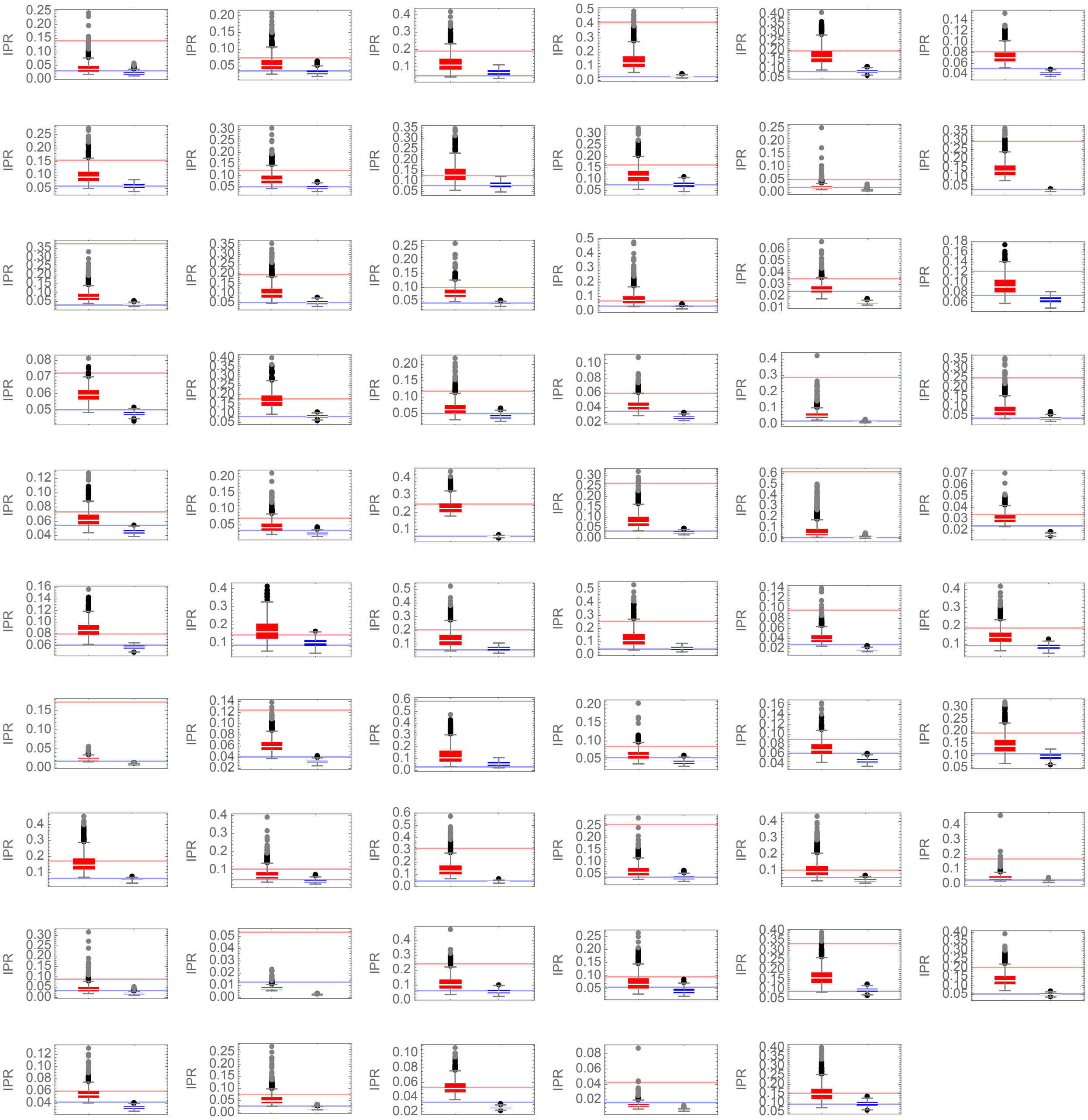

Supplementary Figure 5: **Localization in mutualistic networks: positive strength-degree relation.** Comparison between IPR ($\mathbf{v}_1$ in red, $\mathbf{u}_1$ in blue) for each of the 59 empirical pollinator networks (horizontal solid lines), and Box-Whisker plots for the IPR of the 1000 networks generated through the null model 1 ($\delta = -0.5$).

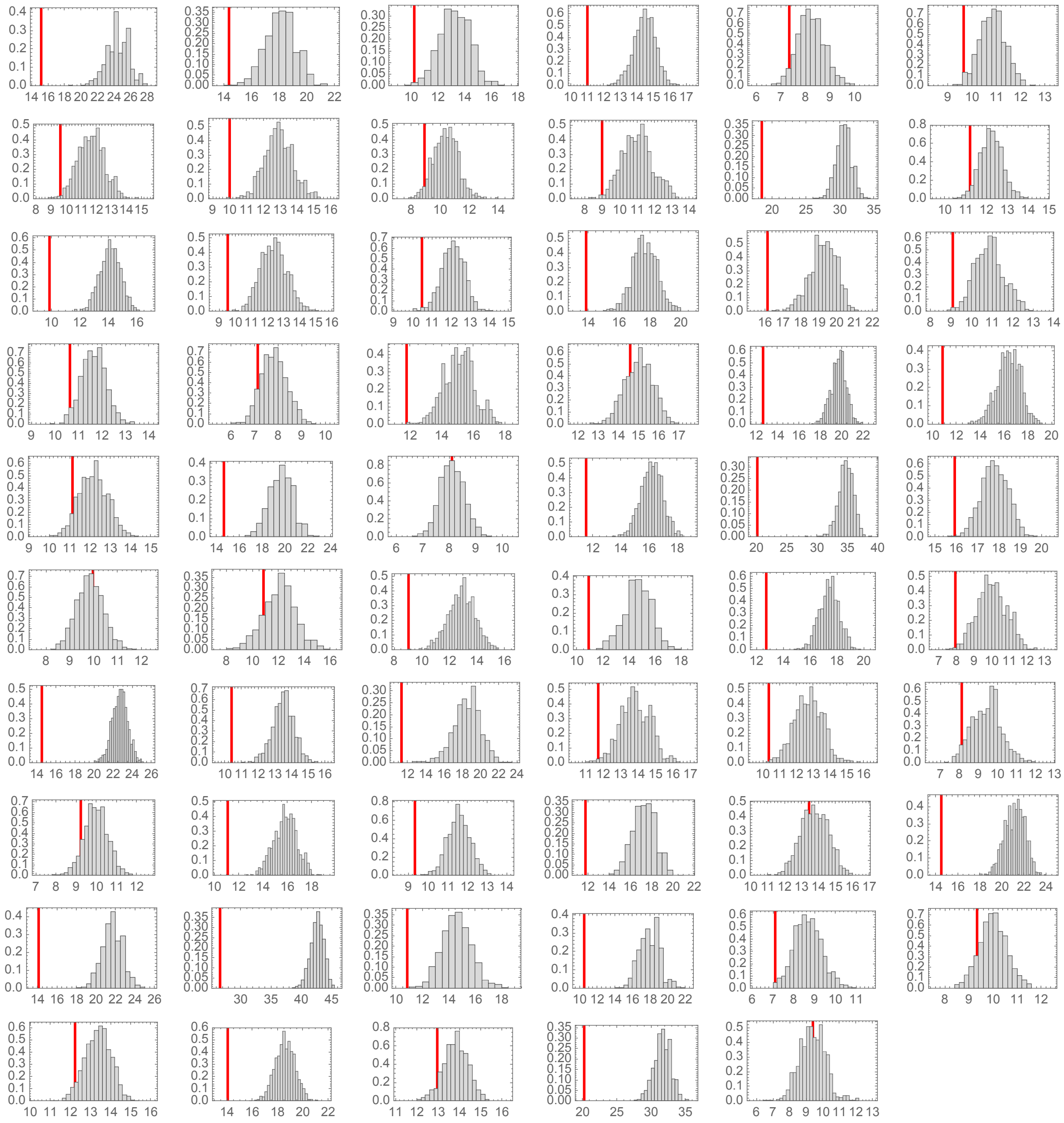

Supplementary Figure 6: **Effect of localization to stability: strength-degree trade-off.** Comparison between $\mathcal{A}_1$ for each of the 59 empirical pollinator networks (vertical red solid lines), and probability distribution (PDF - y axis) of $\mathcal{A}_1^{ran}$ (x axis) obtained from 1000 networks generated through the null model 1 ($\delta = 0.5$).

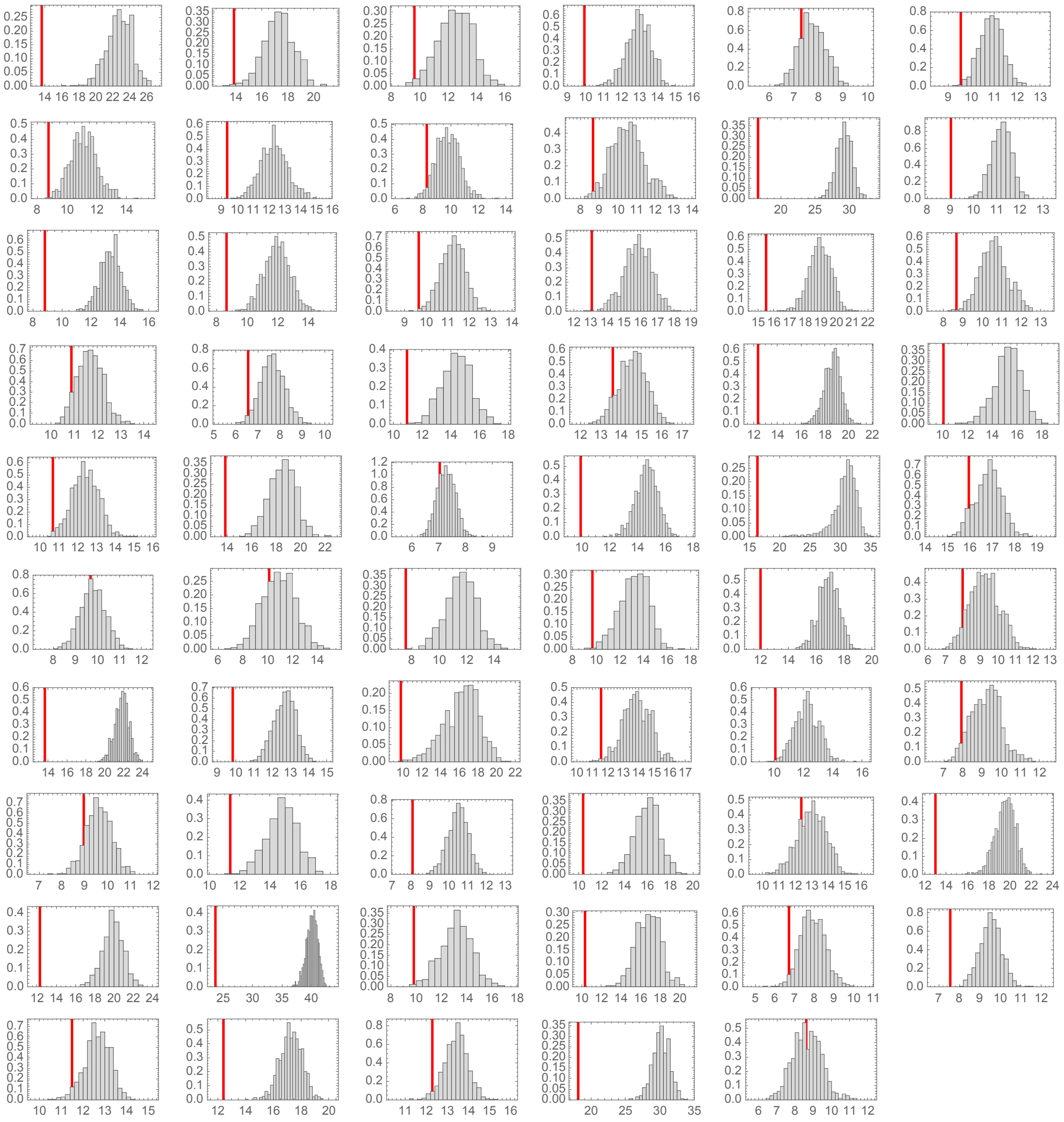

Supplementary Figure 7: **Effect of localization to stability: mean-field case.** Comparison between $\mathcal{A}_1$ for each of the 59 empirical pollinator networks (vertical red solid lines), and probability distribution (PDF - y axis) of $\mathcal{A}_1^{ran}$ (x axis) obtained from 1000 networks generated through the null model 1 ($\delta = 0$).

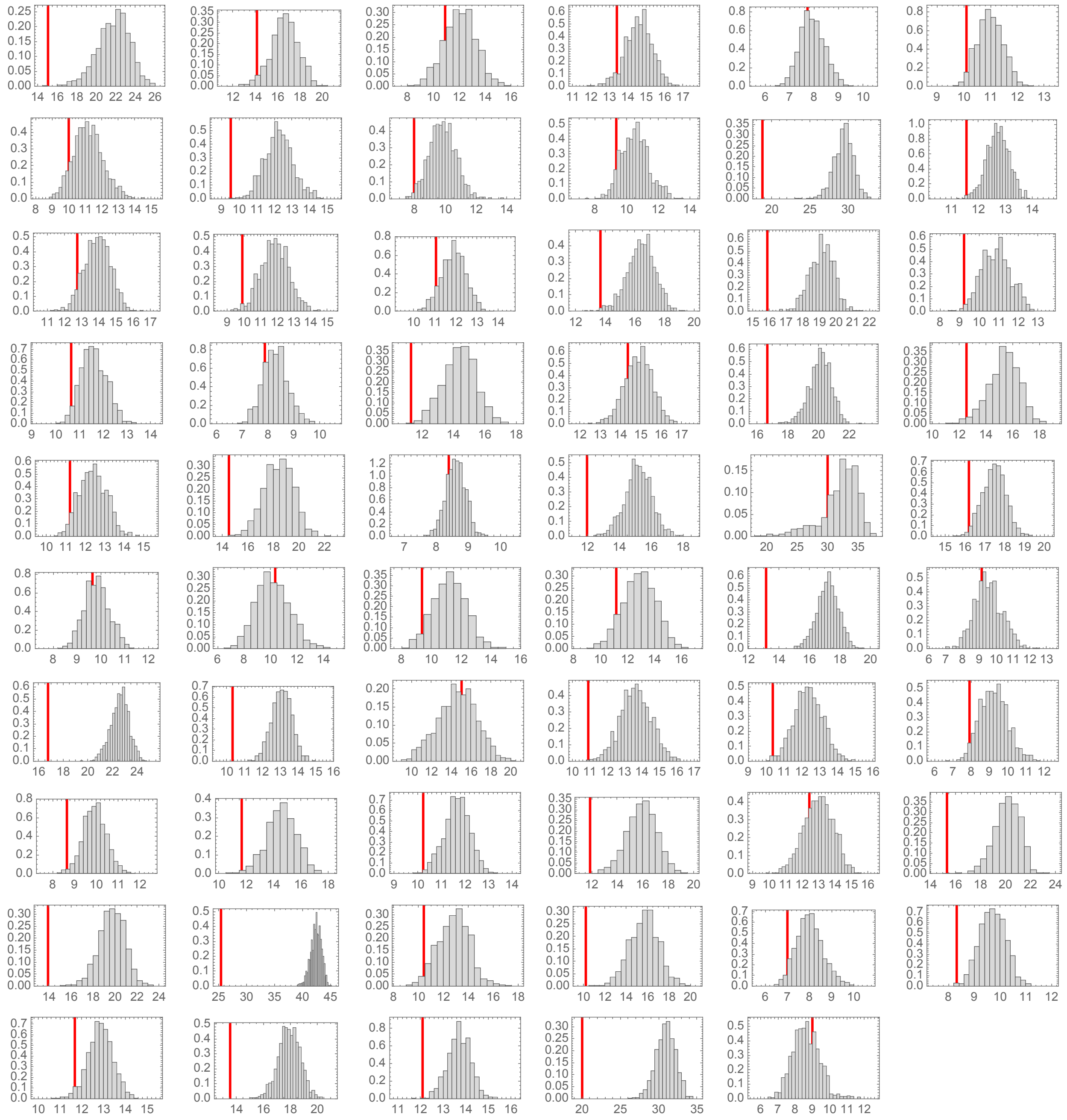

Supplementary Figure 8: **Effect of localization to stability: positive strength-degree relation.** Comparison between $\mathcal{A}_1$ for each of the 59 empirical pollinator networks (vertical red solid lines), and probability distribution (PDF - y axis) of $\mathcal{A}_1^{ran}$ (x axis) obtained from 1000 networks generated through the null model 1 ($\delta = -0.5$).

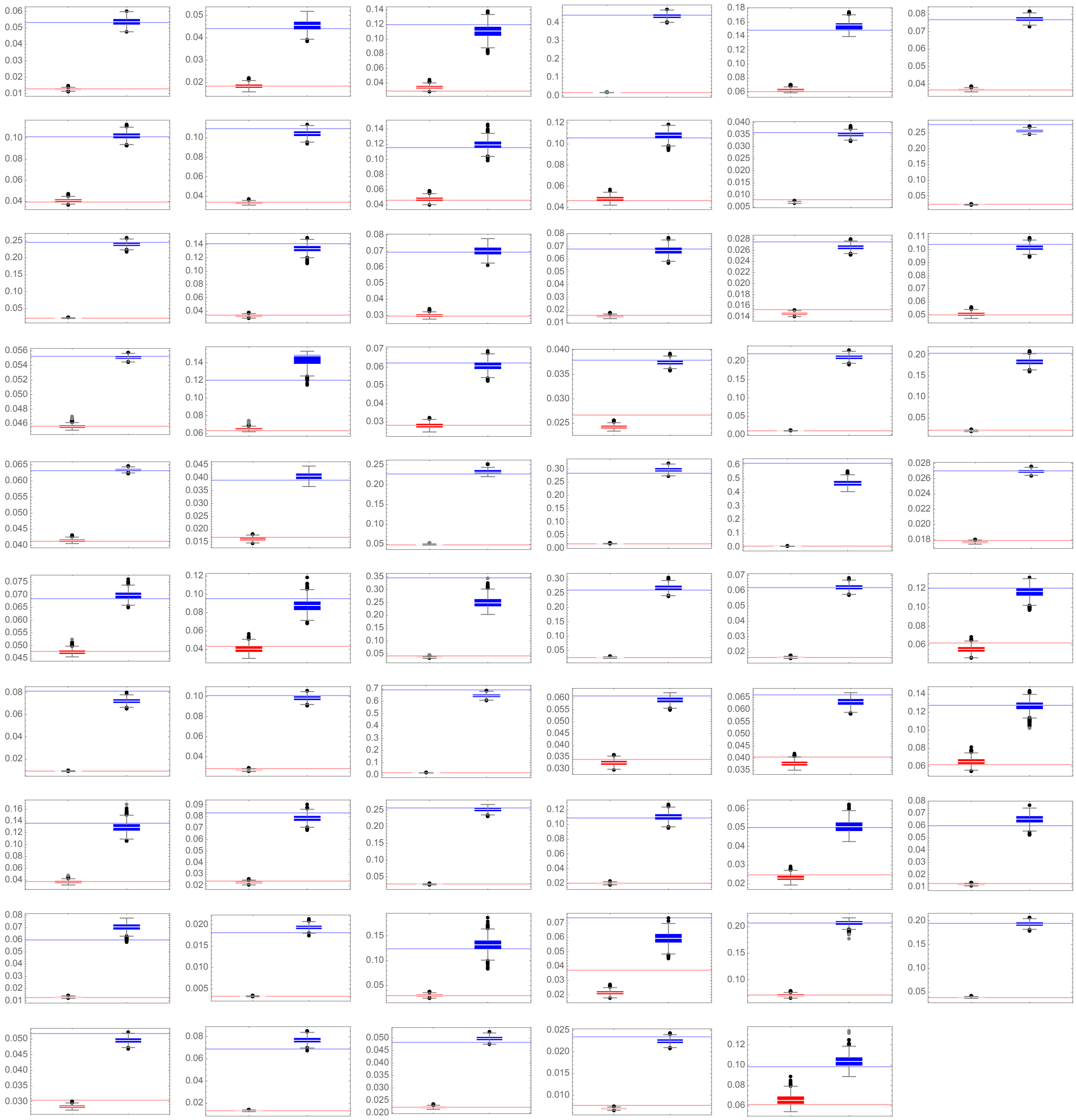

Supplementary Figure 9: **Localization in mutualistic networks: strength-degree trade-off.** Comparison between IPR ($\mathbf{v}_1$ in red, $\mathbf{u}_1$ in blue) for each of the 59 empirical pollinator network (horizontal solid lines), and Box-Whisker plots for the IPR of the 1000 networks generated through the null model 2 ($\delta = 0.5$).

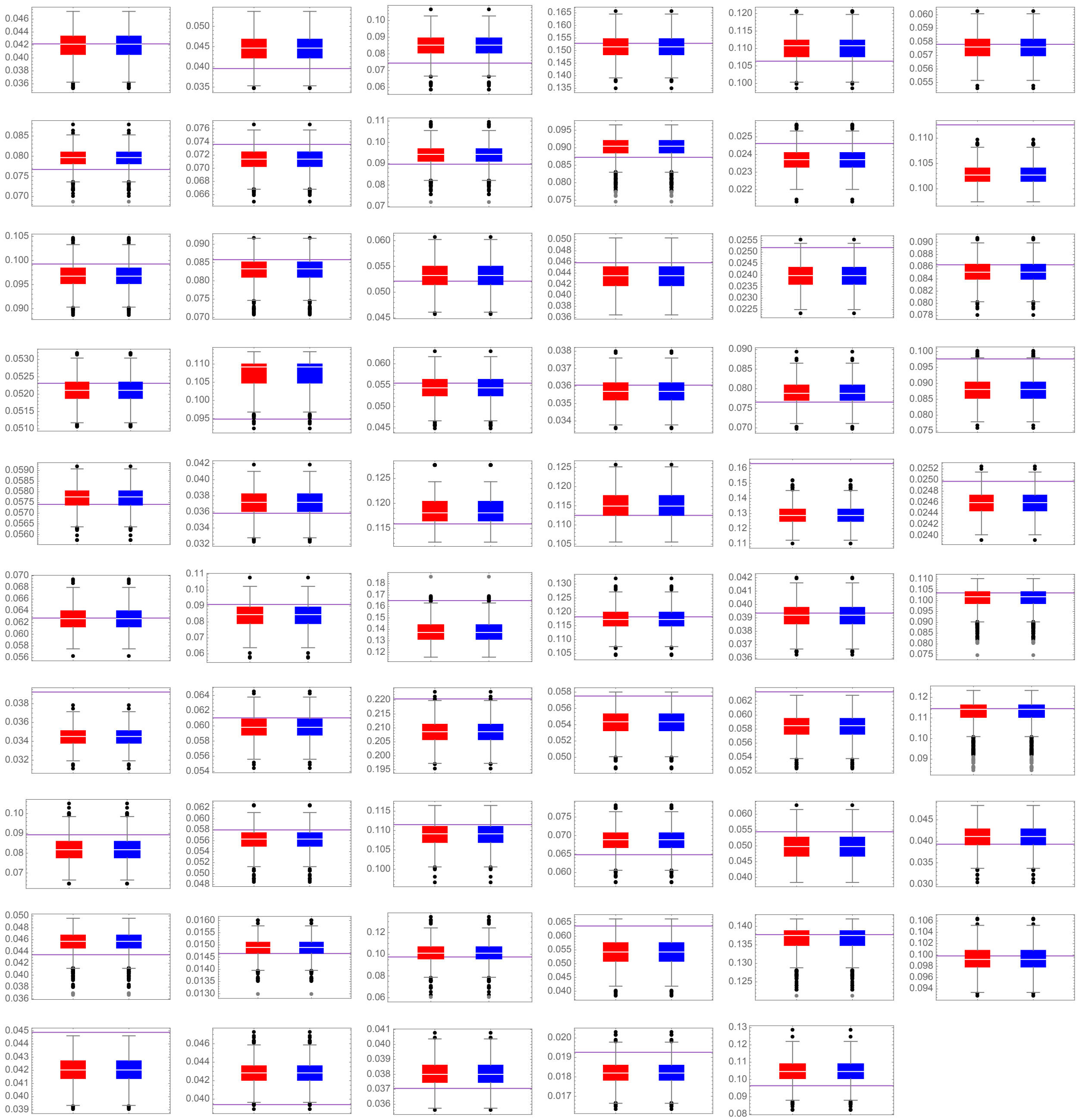

Supplementary Figure 10: **Localization in mutualistic networks: mean field case.** Comparison between IPR ($\mathbf{v}_1$ in red, $\mathbf{u}_1$ in blue) for each of the 59 empirical pollinator network (horizontal solid lines), and Box-Whisker plots for the IPR of the 1000 networks generated through the null model 2 ($\delta = 0$).

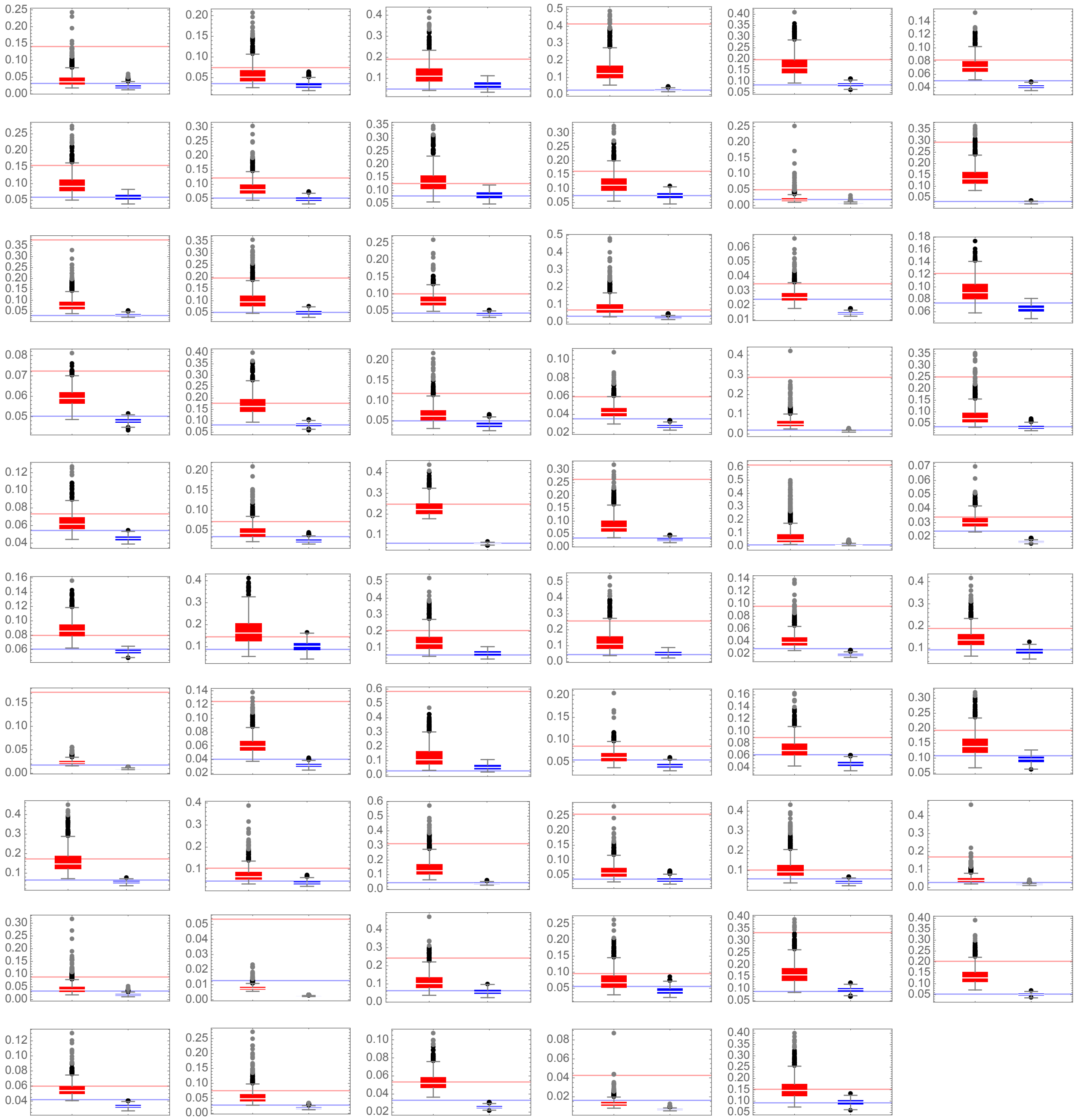

Supplementary Figure 11: **Localization in mutualistic networks: positive strength-degree relation.** Comparison between IPR ($\mathbf{v}_1$ in red, $\mathbf{u}_1$ in blue) for each of the 59 empirical pollinator network (horizontal solid lines), and Box-Whisker plots for the IPR of the 1000 networks generated through the null model 2 ($\delta = -0.5$).

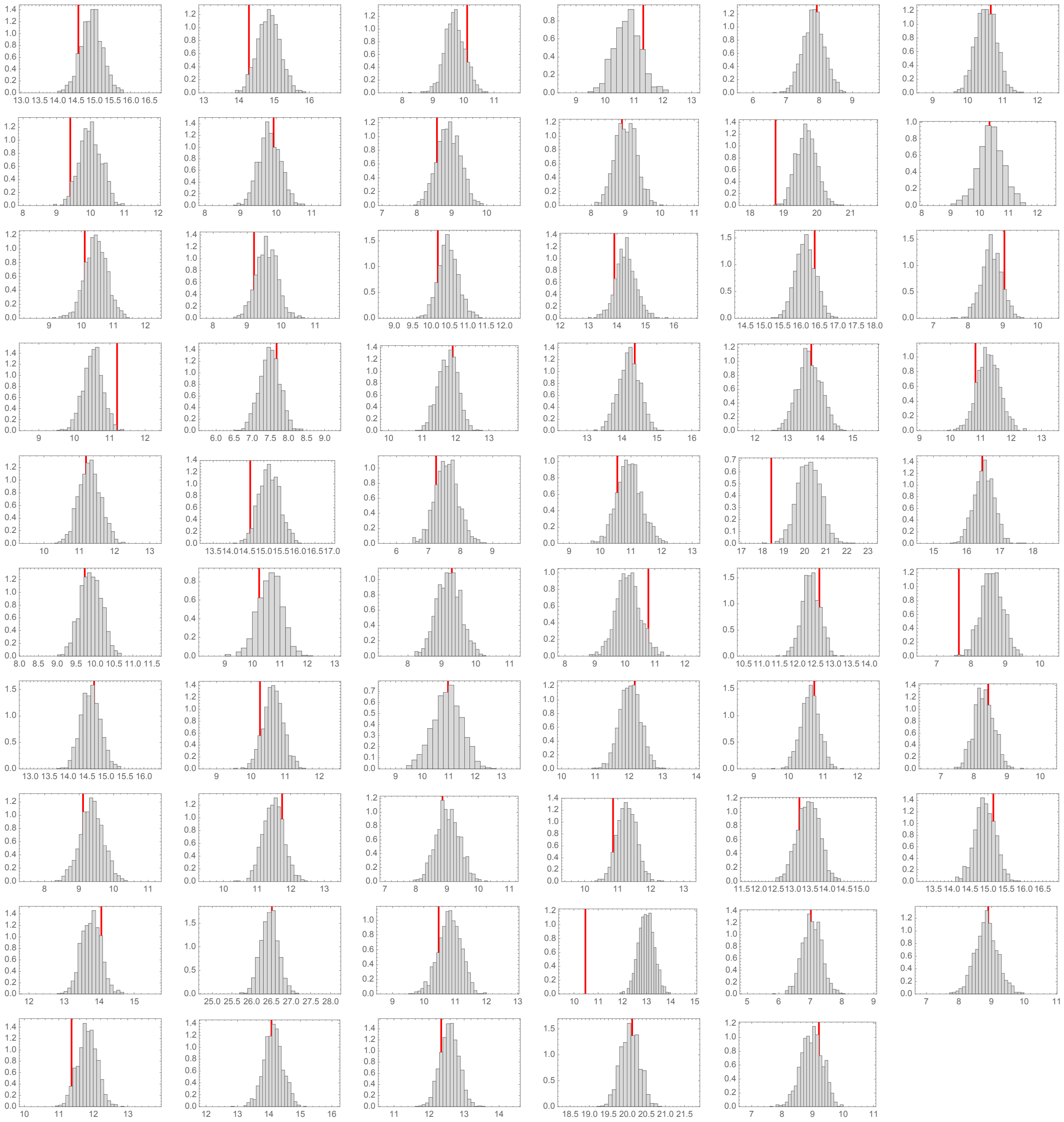

Supplementary Figure 12: **Effect of localization to stability: strength-degree trade-off.** Comparison between $\mathcal{A}_1$ for each of the 59 empirical pollinator network (vertical red solid lines), and probability distribution (PDF - y axis) of $\mathcal{A}_1^{ran}$ (x axis) obtained from 1000 networks generated through the null model 2 ($\delta = 0.5$).

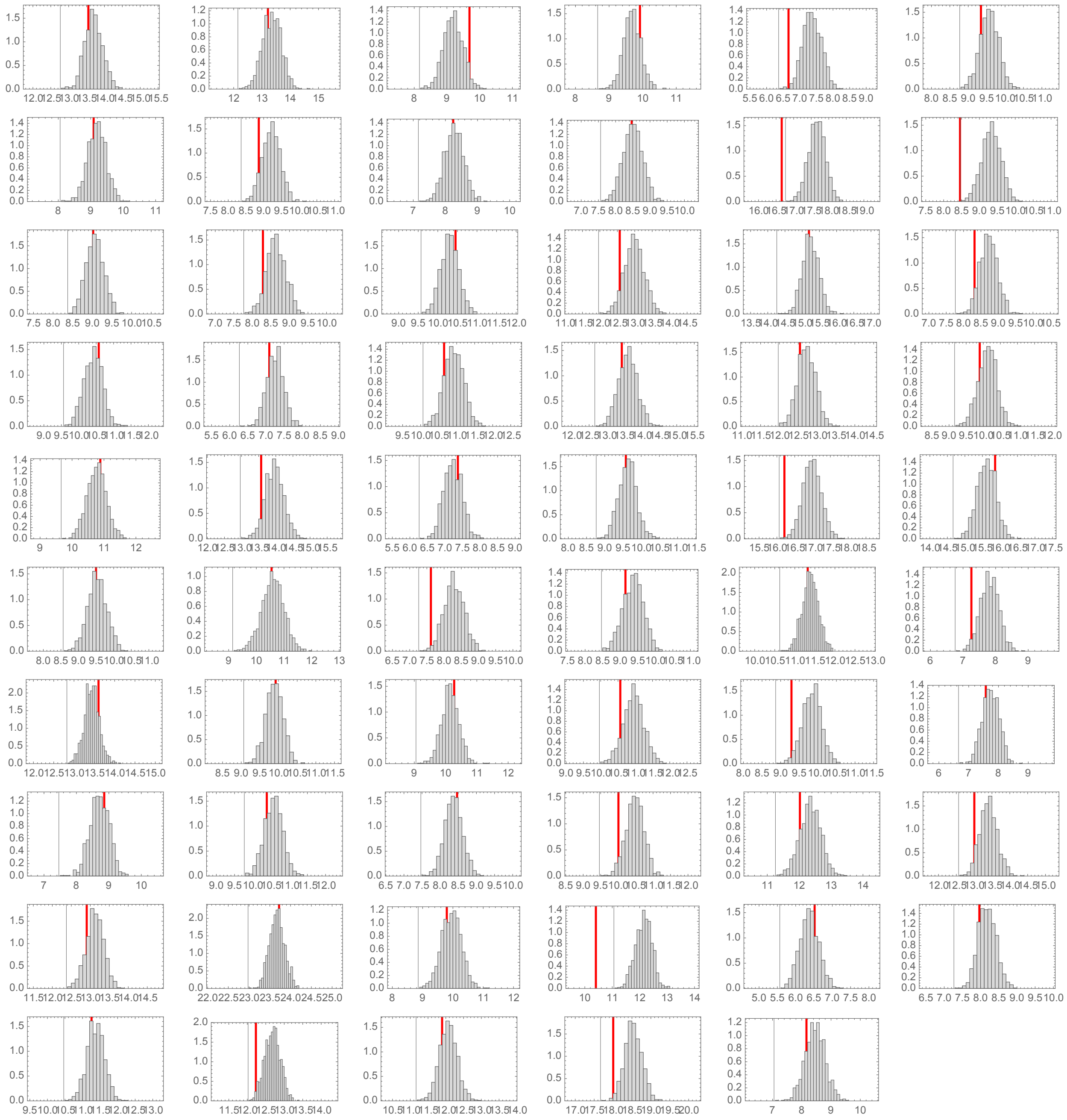

Supplementary Figure 13: **Effect of localization to stability: mean-field case.** Comparison between $\mathcal{A}_1$ for each of the 59 empirical pollinator network (vertical red solid lines), and probability distribution (PDF - y axis) of $\mathcal{A}_1^{ran}$ (x axis) obtained from 1000 networks generated through the null model 2 ($\delta = 0$).

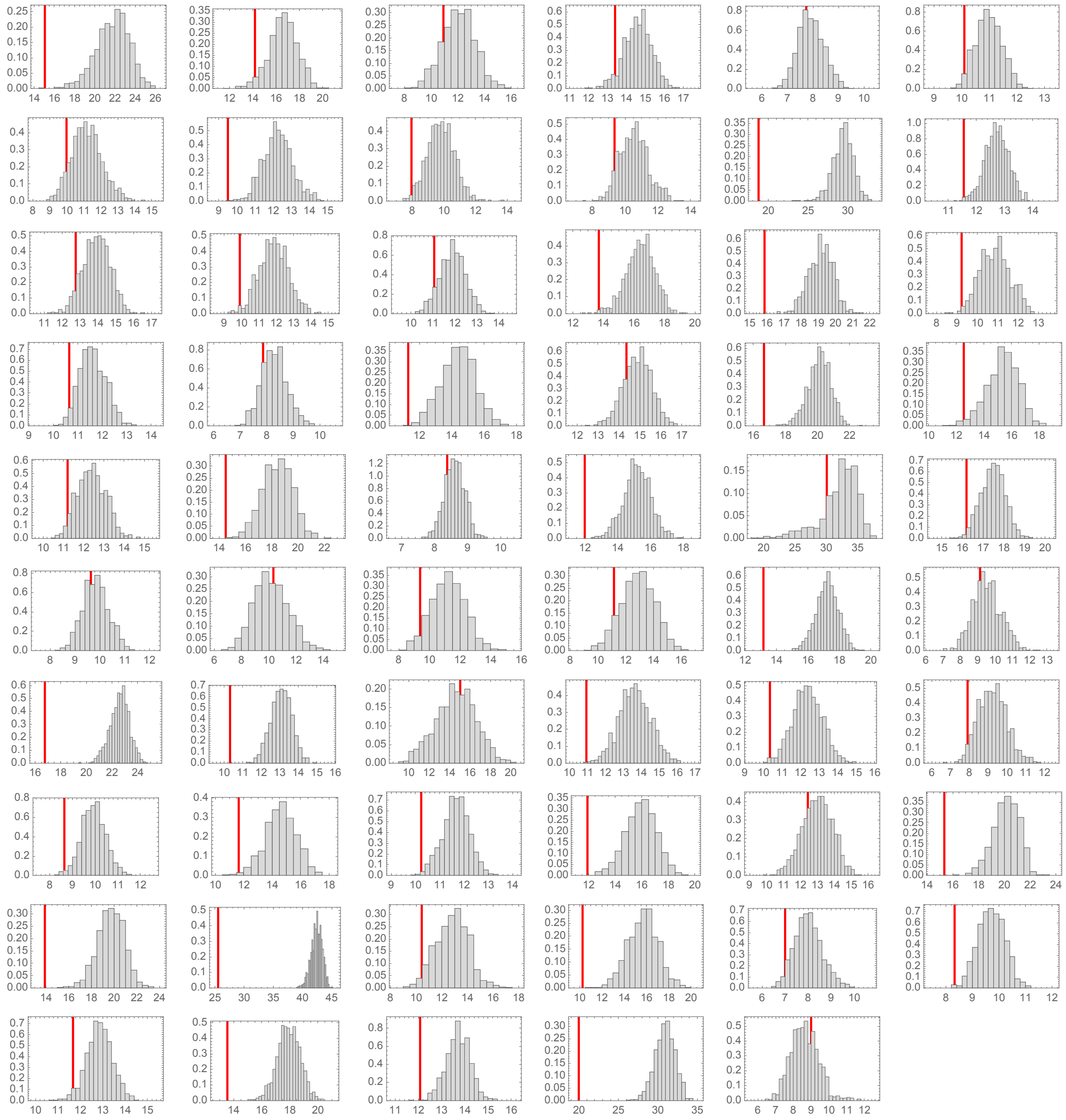

Supplementary Figure 14: **Effect of localization to stability: positive strength-degree relation.** Comparison between $\mathcal{A}_1$ for each of the 59 empirical pollinator network (vertical red solid lines), and probability distribution (PDF - y axis) of $\mathcal{A}_1^{ran}$ (x axis) obtained from 1000 networks generated through the null model 2 ($\delta = -0.5$).

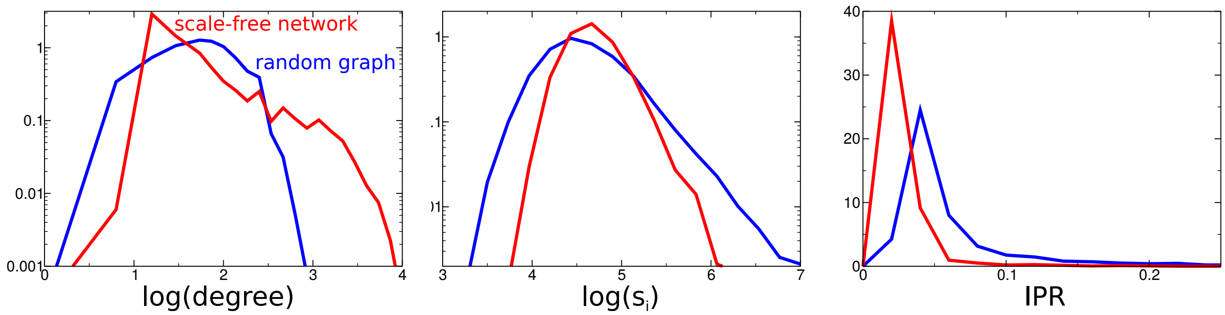

Supplementary Figure 15: **Weighted vs - binary degree distribution.** The degree of localization depends on the distribution of strengths and not on degree distribution. The left panel shows the degree distribution for a random (blue line) and a scale free network (red line). The middle panel shows the strength distribution of the random (blue) and the scale free (red) cases, whereas the last panel shows the corresponding IPR distributions. The anti-nested weights distribution for the scale free network result makes the random network to have a larger IPR than the scale-free network.

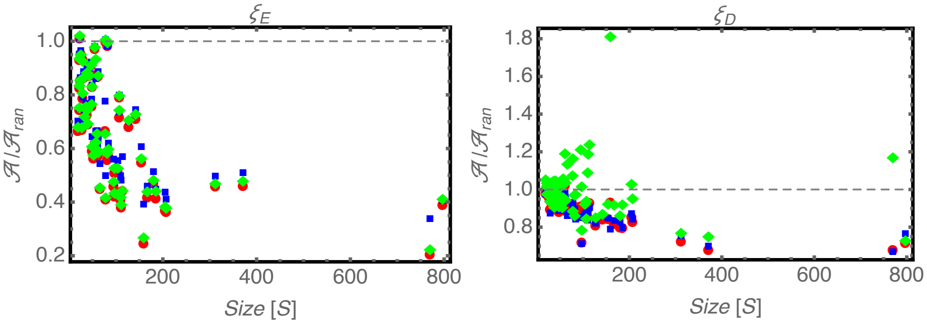

Supplementary Figure 16: **Sensitivity of the results to different type of perturbations.** Effect of the localization on the asymptotic amplitude $\mathcal{A}_1$ of the simulated perturbation ξ_E and ξ_D (with respect to Null Model 2). In most of the cases the perturbation amplitude is attenuated and the attenuation increases for increasing community size. Results are for $\gamma_0(S) = c(\delta)S^{-1}$ (see Supplementary Figure 1).

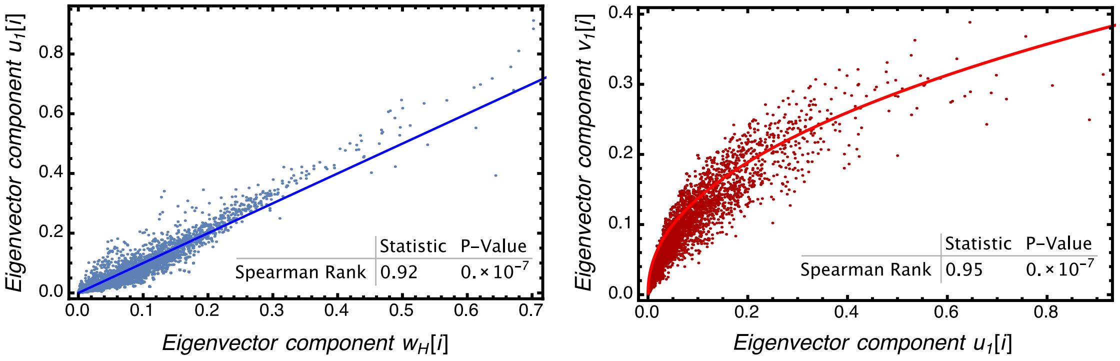

Supplementary Figure 17: **Correlation between eigenvector components.** Spearman correlation test between leading eigenvectors $\{\mathbf{w}_H, \mathbf{u}_1\}$, and $\{\mathbf{u}_1, \mathbf{v}_1\}$. The solid line has been calculated through a non linear fit ax^b.

Supplementary Table 1: **Correlations between network topological and spectral properties under NM1.** Correlations $\rho(x,y)$ measured using Spearman Rank Test (for parametrization $\delta = 0$ using Holling Type I model with a=40 and b=0.05). $rIPR$ refers to null model 1

$\rho(x,y)$	x	y	$P-value$
≈ 0.298	$C \cdot S$	$rIPR[\mathbf{u}_1]$	0.02
≈ 0.249	$C \cdot S$	$rIPR[\mathbf{v}_1]$	0.05
≈ 0.814	S	$rIPR[\mathbf{u}_1]$	$< 10^{-4}$
≈ 0.697	S	$rIPR[\mathbf{v}_1]$	$< 10^{-4}$
≈ 0.807	S	$rIPR[\mathbf{w}_H]$	$< 10^{-4}$
≈ -0.769	S	$\mathcal{A}_1$	$< 10^{-4}$
≈ -0.664	C	$rIPR[\mathbf{u}_1]$	$< 10^{-4}$
≈ -0.571	C	$rIPR[\mathbf{v}_1]$	$< 10^{-4}$
≈ -0.671	C	$rIPR[\mathbf{w}_H]$	$< 10^{-4}$
≈ 0.806	C	$\mathcal{A}_1$	$< 10^{-4}$
≈ 0.761	σ_s^2	$rIPR[\mathbf{u}_1]$	$< 10^{-4}$
≈ 0.628	σ_s^2	$rIPR[\mathbf{v}_1]$	$< 10^{-4}$
≈ -0.477	λ_1	$\mathcal{A}_1$	$< 10^{-4}$
≈ 0.677	λ_1	$rIPR[\mathbf{u}_1]$	$< 10^{-4}$
≈ 0.660	λ_1	$rIPR[\mathbf{v}_1]$	$< 10^{-4}$
≈ 0.635	λ_1	$rIPR[\mathbf{w}_H]$	$< 10^{-4}$

Supplementary Table 2: **Correlations between network topological and spectral properties under NM1.** Correlations $\rho(x, y)$ measured using Sperman Rank Test (parametrization $\delta = -0.5$ and $\gamma_0 = 0.005$ - model independent parametrization).

$\rho(x, y)$	x	y	$P - value$
≈ 0.33	Degree [**k**]	$\mathbf{u}_1$	10^{-4}
≈ 0.62	Strength [**s**]	$\mathbf{u}_1$	10^{-7}
≈ 0.58	Degree [**k**]	$\mathbf{v}_1$	10^{-4}
≈ 0.63	Strength [**s**]	$\mathbf{v}_1$	10^{-7}

Supplementary Table 3: **Correlations between network topological and spectral properties under NM2.** Correlations $\rho(x, y)$ measured using Spearman Rank Test (for parametrization $\delta = 0.5$ using Holling Type I model with a=40 and b=0.05). $rIPR$ refers to null model 2.

$\rho(x, y)$	x	y	$P - value$
≈ 0.207	S	$rIPR[\mathbf{u}_1]$	0.128
≈ 0.200	S	$rIPR[\mathbf{v}_1]$	0.114
≈ 0.200	S	$rIPR[\mathbf{w}_H]$	0.128
≈ -0.338	S	$\mathcal{A}_1$	0.009
≈ -0.152	C	$rIPR[\mathbf{u}_1]$	0.251
≈ -0.215	C	$rIPR[\mathbf{v}_1]$	0.101
≈ -0.192	C	$rIPR[\mathbf{w}_H]$	0.143
≈ 0.806	C	$\mathcal{A}_1$	$< 10^{-4}$

Supplementary Methods

1 Empirical Data and Parametrization using Holling Type I model

Data sets of empirical mutualistic networks used in this work are taken from the Interaction web database and include both insect-flowers and plants-seed dispersal communities (https://www.nceas.ucsb.edu/interactionweb/). We denote the number of animal pollinators by A, and the number of flowers and plants by P ($S = P + A$ is the total number of species in the community). Data are displayed in form of adjacency matrices B indicating presence (B_{ij}=1) or absence (B_{ij}=0) of interactions among species. A possible path to build the community matrix (that we need to study the stability of the system - see Methods in the main text) is to use a specific model for the species population dynamics (i.e. Holling Type I or Holling Type II) (1), and compute the $S \times S$ community matrix Φ describing the ecological community dynamics around one of its stationary stable points. In this case Φ will depend on both the interaction weights (or strenghts) w_{ij}, and the species biomass at equilibrium $\mathbf{x}^*$. As interaction weights are unknown from the data, we parametrize the matrix W describing the interaction strengths so to explore different kinds of ecological scenarios. Specifically we set (assuming no inter-species competition):

$$w_{ij} = \begin{cases} \gamma_0 B_{ij}/k_i^\delta & \text{if } i \in A \text{ and } j \in P \\ -d_i \delta_{ij}^K & \text{otherwise,} \end{cases}$$

where k_i is the degree (number of *mutualistic* partners) of species i, γ_0 is the basal mutualistic strength, δ^K is the Kronecker delta, while d_i describes the species i self-limitation effect (for simplicity here we set d_i=1 $\forall i = 1, ..., S$ and $\gamma_0 = 1.$). $\delta \in [0, 1]$ sets the relationship between mutualistic interaction strength and species degree, and we have investigated different parametrizations corresponding to different ecological scenarios (see Supplementary Figure 1): *A*) Mean field case ($\delta = 0$) where there is no trade-off between species mutualistic strength and species degree (2; 1)); *B*) Interaction strength-degree trade-off ($\delta = 0.5$), where a weak w_{ij} term is associated with a strong w_{ji} term (e.g. a strong dependency of the plant on the pollinator and a weak dependency of the pollinator on the plant) (3; 4); *C*)

Positive strength-degree relation ($\delta = -0.5$), where we set a positive correlation between species interaction strength and degree (corresponding to the structure that optimize species population abundance (1)). The latter scenario is analyzed in detail only in this document. If we model species population dynamics using an Holling Type I (HTI) model, then the elements of the community matrix Φ will simply be

$$\phi_{ij} = x_i^* \cdot w_{ij}, \tag{S1}$$

where $\mathbf{x}^*$ is the vector denoting the stationary species population abundances for the mutualistic community under analysis. We set $\mathbf{x}_i^* \sim \Gamma(x|a, b)$, i.e. a random variable drawn from a Gamma distribution with a shape parameter a and a scale parameter b. Indeed, it has been shown that the Gamma distribution is generally a very good distribution to model the stationary species abundance in ecological community (5). We note that the mean and the variance of the species population abundance within a community are respectively $\langle x^* \rangle = ab$ and $\sigma^2_{\mathbf{x}^*} = ab^2$. If we denote by σ^2_ϕ the variance of the off$-$diagonal elements of the matrix Φ, then by appropriately playing with the parameters we may have two very different scenarios: *a*) $\sigma^2_{\mathbf{x}^*} \gg \sigma^2_\phi$, i.e. the variability within the diagonal elements of Φ is much larger than the variability of the off-diagonal elements; *b*) $\sigma^2_{\mathbf{x}^*} \ll \sigma^2_\phi$, where the opposite is true. In the next section will we show that in the first case, being the community matrix diagonally dominant, both Φ and Φ_{ran} (for any null model - see Methods section in the main text and section 3) are trivially localized. On the other hand, in scenario *b*), the whole topological structure of the interaction network matters, and this is the case we are interested in. Accordingly we set $a = 40$, $b = 0.025$. Using this parametrization, for a fixed δ the stability and reactivity of ecological communities depends on the species self-limitation $\mathbf{d}$. We note that for any given γ_0 it is always possible to find an appropriate $\mathbf{d}$ so that Φ has all negative real parts eigenvalues without affecting the structure of the leading eigenvectors corresponding to the eigenvalue with the largest real part λ_1 (see Methods in the main text).

1.1 Weighted Empirical Network

Regarding the empirical networks, we have used only binary interactions networks, as what is actually measured in weighted empirical networks are the number of visits between pollinators and plants species, and not the interaction strengths. In order to transform species visits data in interaction

strengths, there is a non trivial and not unique theoretical machinery to use. In fact, the available weighted data on mutualistic interactions are based on counting the number of visits. This matrix of visits reflects the structure of the actual interaction network, but the actual interaction strength are not, at least in principle, simply related to the frequency of visits. Obtaining the interaction strength from these visitation data can be still considered an open problem and cannot be solved in an assumption-free setting (6). Different approaches and assumptions could correspond to very different interaction patterns (7; 8), that reflects in a non-trivial way on localization. The approaches we followed in the manuscript are based on very mild and controllable assumptions. Therefore, to avoid adding unnecessary complexity and uncertainty to the outputs of our study, we have preferred the line of using binary interaction matrix (called B) with a classical parameterization (9; 10; 11) that allows the exploration of several different ecological scenarios.

2 Random Matrix Approach

In order to test the generality of our results, we also perform the analysis presented in the main text (where Φ is built as described in the section above), also using a minimalist general theoretical framework independent of any specific dynamics (i.e. Holling Type I or Holling Type II) (12; 13). In this case we directly compute the $S \times S$ community matrix Φ from the adjacency matrix B by using a random matrix approach (12; 13). Specifically we set

$$\phi_{ij} = \begin{cases} \gamma_0 B_{ij}/k_i^\delta & \text{if } i \in A \text{ and } j \in P \\ -d_i \delta_{ij}^K & \text{otherwise,} \end{cases}$$

where this time both d_i and γ_0 are random variables (e.g. Gaussian) peaked around $\bar{d}$ and $\bar{\gamma}$, respectively.

We found that results for this case are qualitatively the same of those obtained by calculating the community matrix through an HTI model.

3 Null Models

All results are compared to two corresponding null models, so to understand the net effect of the network architecture on the stability of the mutualistic ecological communities.

3.1 Null Model 1

In the main text, all results refer to Null Model 1, where the random community matrix Φ_{ran} (see Methods in main text) is built by assigning at random the same number of links observed in the corresponding real mutualistic network, while keeping the network connected. In this way we have one realization of the random adjacency binary matrix B_{ran}. For each empirical matrix we generated 1000 randomized matrices. We then achieve Φ_{ran} as described for real empirical networks in section 1.

3.2 Null Model 2

Randomizations with fixed degree distribution. We randomize the networks conserving exactly the degree distribution (i.e. the row and column sum of the bipartite block of the binary incidence matrix). For each empirical matrix we generated 1000 randomized matrices using the curveball algorithm (14). The algorithm is based on random moves that swaps the links in the networks conserving the degree and require to specify the number of moves after which the randomization is consider completed. In the original algorithm the authors suggested to run the simulation for at least 5*min(P,A) times ($S = P + A$ is the total number of species in the community), but we ran them for 100*min(P,A) times to guarantee the convergence.

4 Detailed Results on Localization and its Effect on Perturbations Propagation.

In this section we present the localization patterns for each empirical networks and their effect on the asymptotic perturbation amplitudes compared to networks generated by null model 1 (as shown in main text and here in section 5) and null model 2, which generates networks with localization patterns similar to those observed in empirical pollinator networks. Following the methods section presented in the main text, $\mathbf{v}_1$, $\mathbf{u}_1$ represent the leading right/left eigenvector corresponding to λ_1, the eigenvalue of Φ with the highest real part. $\mathbf{w}_H$ is the leading eigenvector corresponding to λ_H, the highest eigenvalue of the symmetric reactive matrix ($H = (\Phi + \Phi^T)/2$). We measure the localizatoin using the inverse participation ratio IPR (15), i.e., $IPR = \sum_{i=1}^{S} q_1(i)^4 / (\sum_{i=1}^{S} q_1(i)^2)^2$, where $\mathbf{q}_1 = \mathbf{v}_1$, $\mathbf{u}_1$ or $\mathbf{w}_H$. In par-

ticular, we identify localization patterns by computing the $rIPR$, i.e. the ratio between the IPR of each real empirical network and the IPR of the corresponding random null model: $rIPR_i = IPR_i/\langle IPR_i^{ran}\rangle$. The average $\langle\cdot\rangle$ is taken among 1000 realizations of Φ_{ran}. If $rIPR$ is significantly larger than one, then the system is localized. Otherwise we say that the system is not localized. Finally the amplitude associated with the asymptotic propagation of the perturbation through the ecological network is defined as $\mathcal{A}_1 = (\mathbf{u}_1 \cdot \xi)/(\mathbf{v}_1 \cdot \mathbf{u}_1)$ (see Eq. (1) in the main text and methods section). In all cases we set $d = 1$, and we chose the parameters of the Gamma distribution so to have an average species abundance of $\langle x\rangle = 1$ and variance $\sigma_x^2 < \sigma_\phi^2$ (in particular we set $a = 40$, $b = 0.025$ and $\gamma_0 = 1$). We thus obtain 59 community matrices Φ (one for each empirical pollinator network) for each of the three different ecological scenarios $\delta = 0.5$, $\delta = 0.$ and $\delta = -0.5$.

4.1 Null Model 1

As shown in Figures S2, localization patterns for $\delta = -0.5$ are qualitatively the same of those presented in the main text (Figure 2a-c) for $\delta = 0$ and $\delta = 0.5$.

Figures S3-S5 show the statistics of the localization patterns for each of the 59 networks and for different parametrizations through the Box-Whisker plots. The ends of the whiskers represent the minimum and maximum, whereas the ends of the box are the first and third quartiles and the white bar denotes the median. Single points represent outliers realizations. We finally study the effect of localization of the attenuation of the asymptotic amplitude $\mathcal{A}_1$. For each of the 59 networks, and for the different parametrization we calculate $\mathcal{A}_1$ and compare it to the distribution of $\mathcal{A}_1^{ran}$ for 1000 random networks generated by null model 1. As Figures S6-8 show, localization indeed attenuates the perturbation amplitude ($\mathcal{A}_1 < \mathcal{A}_1^{ran}$).

4.2 Null Model 2

As already shown in the main text, null model 2 generate most of the times random networks that have similar localization patterns to those observed in empirical pollinator networks. These results suggest that it is the heterogeneous degree distribution of empirical systems (16) the responsible of their higher localization: once we constraint the degree distributions to be fixed, then null model 2 generates very similar localization patterns of those

observed in empirical mutualistic networks. Nodes weighted degrees (or strength s_i - see below) also play a crucial role. In fact, a network with binary core-pheriperhy structure, but having anti-nested distributed weights (8), will not be localized because, contrarily to its degree distribution, the weighted degree distribution will be homogeneous (see section below). Also in this case we study the effect of localization of the attenuation of the asymptotic amplitude $\mathcal{A}_1$ for each of the 59 networks, and for the different parametrization we calculate $\mathcal{A}_1$ and compare it to the distribution of $\mathcal{A}_1^{ran}$ for 1000 random networks generated by null model 2. As Figures S12-14 show, as a consequence of the fact that the null mode 2 generate similar localization patterns to those observed in real data, then we find no significant attenuation of the asymptotic amplitude ($\mathcal{A}_1 \approx \mathcal{A}_1^{ran}$).

Figures S9-S11 show the statistics of the localization patterns for each of the 59 networks and for different parametrizations through the Box-Whisker plots with respect null model 2. Only very few empirical networks are localized with respect this null model.

Finally we test if the correlation between the $rIPR$ calculated through null model 1 and the network topological properties shown in the main text (Figure 4) and Table 2, still hold when we calculate the $rIPR$ using null model 2. As Supplementary Table 3 shows, we found that the correlations between rIPR (calculated through null model 2), size S and connectance C are now not significant, and thus we can conclude that it is indeed the heterogeneity in the weighted degree distribution the key structural aspect of ecological networks that is related to localization.

5 Crucial role of the strength distribution.

In this section we show that, when considering weighted networks, what determines localization of the leading eigenvector(s) is not necessarily the degree distribution, but rather the distribution of nodes strengths ($s_i = \sum_j w_{ij}$). We consider two different networks: a random networkk (Erdös-Renyi (17)) and a scale-free (Barabasi-Albert (18)) network, with same size S and number of links L. The second network is much more nested (as a consequence of its heterogeneous degree distribution) than the first one. However, we arranged the weights in such a way that the random network has a larger variance in the strengths with respect to the heterogeneous network. We call anti-nested(8) this arrangement of weights (the binary matrix is nested, while

the weighted matrix is less nested than its random counterpart). In this case the scale-free nested network displays a lower localization than the random network (i.e. a network with random connection and weights drawn from the same distribution). An example of this result is shown in Supplementary Figure 15.

6 Results for different type of perturbations.

We here present here the results of the effect of localization on stability (in terms of the asymptotic amplitude of the perturbation $\mathcal{A}_1$) for two type of perturbations: a) A noise ξ_D that is independent of species characteristics. In particular, we model ξ_D as drawn from a normal distribution $\mathcal{N}(1, \zeta)$. b) A noise ξ_E that is species dependent, i.e. proportional to each of the species degree ($\xi_E(i) \propto k_i \xi_D(i)$). For simplicity, in this and in the following sections we used the model independent parametrization (see section 2). Results are robust with respect to different parametrizations of the community matrix.

7 Which species are more localized?

In this section, we study the topological properties of the nodes (species) which have the largest components of the eigenvectors. To accomplish this, we quantify the number of localized species by setting a threshold θ (node i is localized if $v_1(i)(u_1(i)) > \theta$) and look at the centrality properties of these nodes. The centrality of a node, is a measure that assigns a given "importance" to that node in the network (17). There are several ways to denote this importance. The simplest one is the degree centrality k_i, the number of connections that a node i has. But one may want to consider also "the quality" of the neighbors of a given node, not just its number. Then the eigenvector centrality has been introduced (17), where the centrality of node i is given by the i-th component of the principal eigenvector (i.e. relative to the maximum eigenvector of the graph matrix). We set $\theta = 1.5/\sqrt{S}$ ($v_1(i) \sim u_1(i) = 1/S$ would correspond to the extended, non localized case). We also compute the PageRank (PRC) measure, which is used by the Google web search corporation as a central part of their web ranking technology (17). We note that all the centrality measures are calculated on the symmetric (binary) adjacency matrix, and thus do not depend on the parametrization

of the ecological networks. In all cases we find that the localized nodes are on average the most central nodes of the networks, i.e. nodes belonging to the core of the core-periphery (nested) structure of the mutualistic species interaction networks. These results suggest that the peripheral nodes, i.e. the specialized species, are more "protected" by perturbations affecting the system dynamics. The full statistic of the correlation among these quantities for the parametrization $\delta = 0.$ can be found in Table 1 (results for $\delta = -0.5$ are qualitatively the same - see Table 2).

Both pair of vectors $\{\mathbf{w}_H, \mathbf{u}_1\}$ and $\{\mathbf{u}_1, \mathbf{v}_1\}$ are strongly correlated (see Supplementary Figure 17), indicating the ability of the network to contain the spread of the perturbation within the network, i.e., those species that are more affected by the perturbation at time $t \to 0^+$ (components i with large $w_H(i)$), are those (or very close) to those that are most hit by the perturbation asymptotically (components i with large $u_1(i)$), that in turn are those species that are most affected asymptotically by the perturbation after its spread through the network (components i with large $v_1(i)$).

References

[1] Suweis, S., Simini, F., Banavar, J. R. & Maritan, A. Emergence of structural and dynamical properties of ecological mutualistic networks. *Nature* **500**, 449–52 (2013). URL `http://www.ncbi.nlm.nih.gov/pubmed/23969462`.

[2] Bastolla, U. *et al.* The architecture of mutualistic networks minimizes competition and increases biodiversity. *Nature* **458**, 1018–20 (2009). URL `http://www.ncbi.nlm.nih.gov/pubmed/19396144`.

[3] Guimarães, P. R., Rico-Gray, V., Dos Reis, S. F. & Thompson, J. N. Asymmetries in specialization in ant-plant mutualistic networks. *Proceedings of the Royal Society B: Biological Sciences* **273**, 2041–2047 (2006).

[4] Guimaraes Jr, P. R. *et al.* Interaction intimacy affects structure and coevolutionary dynamics in mutualistic networks. *Current Biology* **17**, 1797–1803 (2007).

[5] Azaele, S. *et al.* Towards a unified descriptive theory for spatial ecology: predicting biodiversity patterns across spatial scales. *Methods in Ecology and Evolution* (2014).

[6] Vázquez, D. P., Ramos-Jiliberto, R., Urbani, P. & Valdovinos, F. S. A conceptual framework for studying the strength of plant–animal mutualistic interactions. *Ecology letters* **18**, 385–400 (2015).

[7] Bascompte, J., Jordano, P. & Olesen, J. M. Asymmetric coevolutionary networks facilitate biodiversity maintenance. *Science* **312**, 431–433 (2006).

[8] Staniczenko, P. P., Kopp, J. C. & Allesina, S. The ghost of nestedness in ecological networks. *Nature communications* **4**, 1391 (2013).

[9] Saavedra, S. & Bascompte, J. Structural stability. *Science* **to be published** (2014).

[10] Rohr, R. P., Saavedra, S. & Bascompte, J. On the structural stability of mutualistic systems. *Science* **345**, 1253497 (2014).

[11] Suweis, S., Grilli, J. & Maritan, A. Disentangling the effect of hybrid interactions and of the constant effort hypothesis on ecological community stability. *Oikos* **123:**, 525532 (2014). URL `http://doi.wiley.com/10.1111/j.1600-0706.2013.00822.x`.

[12] May, R. M. Will a Large Complex System be Stable? *Nature* **238**, 413–414 (1972). URL `http://www.nature.com/doifinder/10.1038/238413a0`.

[13] Allesina, S. & Tang, S. Stability criteria for complex ecosystems. *Nature* (2012). URL `http://www.ncbi.nlm.nih.gov/pubmed/22343894`.

[14] Strona, G., Nappo, D., Boccacci, F., Fattorini, S. & San-Miguel-Ayanz, J. A fast and unbiased procedure to randomize ecological binary matrices with fixed row and column totals. *Nature communications* **5** (2014).

[15] Lee, P. A. & Ramakrishnan, T. Disordered electronic systems. *Reviews of Modern Physics* **57**, 287 (1985).

[16] Montoya, J. M., Pimm, S. L. & Solé, R. V. Ecological networks and their fragility. *Nature* **442**, 259–64 (2006). URL `http://www.ncbi.nlm.nih.gov/pubmed/16855581`.

[17] Newman, M. *Networks: an introduction* (Oxford University Press, 2010).

[18] Albert, R. & Barabási, A.-L. Statistical mechanics of complex networks. *Reviews of Modern Physics* **74**, 47–97 (2002). URL `http://link.aps.org/doi/10.1103/RevModPhys.74.47`.
